# Lys-63-linked Ubiquitination of γ-Aminobutyric Acid (GABA), Type B1, at Multiple Sites by the E3 Ligase Mind Bomb-2 Targets GABA_B_ Receptors to Lysosomal Degradation*

**DOI:** 10.1074/jbc.M116.750968

**Published:** 2016-08-29

**Authors:** Khaled Zemoura, Claudia Trümpler, Dietmar Benke

**Affiliations:** From the ‡ Institute of Pharmacology and Toxicology, University of Zurich,; the § Neuroscience Center Zurich, University of Zurich and ETH Zurich, and; the ¶ Drug Discovery Network Zurich (DDNZ), Winterthurerstrasse 190, CH-8057 Zurich, Switzerland

**Keywords:** E3 ubiquitin ligase, GABA receptor, lysosome, protein degradation, ubiquitylation (ubiquitination)

## Abstract

GABA_B_ receptors are heterodimeric G protein-coupled receptors, which control neuronal excitability by mediating prolonged inhibition. The magnitude of GABA_B_ receptor-mediated inhibition essentially depends on the amount of receptors in the plasma membrane. However, the factors regulating cell surface expression of GABA_B_ receptors are poorly characterized. Cell surface GABA_B_ receptors are constitutively internalized and either recycled to the plasma membrane or degraded in lysosomes. The signal that sorts GABA_B_ receptors to lysosomes is currently unknown. Here we show that Mind bomb-2 (MIB2)-mediated Lys-63-linked ubiquitination of the GABA_B1_ subunit at multiple sites is the lysosomal sorting signal for GABA_B_ receptors. We found that inhibition of lysosomal activity in cultured rat cortical neurons increased the fraction of Lys-63-linked ubiquitinated GABA_B_ receptors and enhanced the expression of total as well as cell surface GABA_B_ receptors. Mutational inactivation of four putative ubiquitination sites in the GABA_B1_ subunit significantly diminished Lys-63-linked ubiquitination of GABA_B_ receptors and prevented their lysosomal degradation. We identified MIB2 as the E3 ligase triggering Lys-63-linked ubiquitination and lysosomal degradation of GABA_B_ receptors. Finally, we show that sustained activation of glutamate receptors, a condition occurring in brain ischemia that down-regulates GABA_B_ receptors, considerably increased the expression of MIB2 and Lys-63-linked ubiquitination of GABA_B_ receptors. Interfering with Lys-63-linked ubiquitination by overexpressing ubiquitin mutants or GABA_B1_ mutants deficient in Lys-63-linked ubiquitination prevented glutamate-induced down-regulation of the receptors. These findings indicate that Lys-63-linked ubiquitination of GABA_B1_ at multiple sites by MIB2 controls sorting of GABA_B_ receptors to lysosomes for degradation under physiological and pathological conditions.

## Introduction

The number of neurotransmitter receptors at the cell surface available for signaling in neurons needs to be precisely tuned to a given cellular state and consequently must be dynamically adjusted to altered conditions. One key player regulating their amount as well as their life span is protein degradation. Two major cellular protein degradation systems control the number of neurotransmitter receptors, lysosomes and proteasomes. Interestingly, both systems rely on ubiquitination as a signal that tags most membrane proteins for degradation. For proteasomal degradation, primarily Lys-48-linked polyubiquitination is required, and for lysosomal degradation, primarily Lys-63-linked polyubiquitination is required (1). Both degradation pathways are involved in the regulation of G protein-coupled GABA_B_ receptors. GABA_B_ receptors are heterodimers assembled from GABA_B1_ and GABA_B2_ subunits and are activated by γ-aminobutyric acid (GABA), the main inhibitory neurotransmitter in the brain, to regulate excitability of neurons. At presynaptic locations, GABA_B_ receptors suppress neurotransmitter release mainly by inhibiting voltage-gated Ca^2+^ channels, whereas at postsynaptic sites they induce slow inhibitory postsynaptic currents by activating Kir3-type K^+^ channels (2). GABA_B_ receptors are involved in the regulation of all main brain functions ranging from synaptic plasticity (3), neuronal network activity (4, 5), to neuronal development (6).

An important factor regulating GABA_B_ receptor signaling is the dynamic control of their cell surface expression via protein degradation. So far, the following two mechanisms have been identified: 1) proteasomal degradation of the receptors in the endoplasmic reticulum (ER),^2^ and 2) lysosomal degradation of receptors internalized from the plasma membrane. The amount of GABA_B_ receptors in the ER available for forward trafficking to the cell surface is determined by the rate of their proteasomal degradation via the ER-associated degradation (ERAD) machinery (7). Proteasomal degradation of ER-residing GABA_B_ receptors is regulated by the activity state of the neuron via Lys-48-linked ubiquitination of GABA_B2_ and requires interaction of the GABA_B2_ C terminus with the proteasomal AAA-ATPase Rpt6 (7, 8). In contrast, GABA_B_ receptors at the cell surface are constitutively endocytosed and either recycled to the plasma membrane or degraded in lysosomes (9–13). Lysosomal degradation of GABA_B_ receptors is most likely mediated via the ESCRT (endosomal sorting complex required for transport) machinery (13), which sorts ubiquitinated membrane proteins to lysosomes (14). Interestingly, USP14 (ubiquitin-specific protease 14) has been implicated in sorting ubiquitinated GABA_B_ receptors to lysosomal degradation (15). Lysosomal degradation of GABA_B_ receptors appears to be tightly regulated because excessive activity of glutamate receptors, a condition occurring in brain ischemia, rapidly down-regulates GABA_B_ receptors by preferential sorting them to the lysosomal degradation pathway at the expense of receptor recycling (16–19). The specific signal(s) that sorts GABA_B_ receptors to lysosomal degradation under normal as well as pathological conditions is currently unknown. Here we show that Lys-63-linked ubiquitination by the E3 ligase mindbomb-2 (MIB2) of GABA_B1_ at multiple sites targets GABA_B_ receptors to the lysosomal degradation pathway.

## Results

### 

#### 

##### Lysosomal Degradation Regulates Cell Surface Expression of GABA_B_ Receptors

**FIGURE 1.**
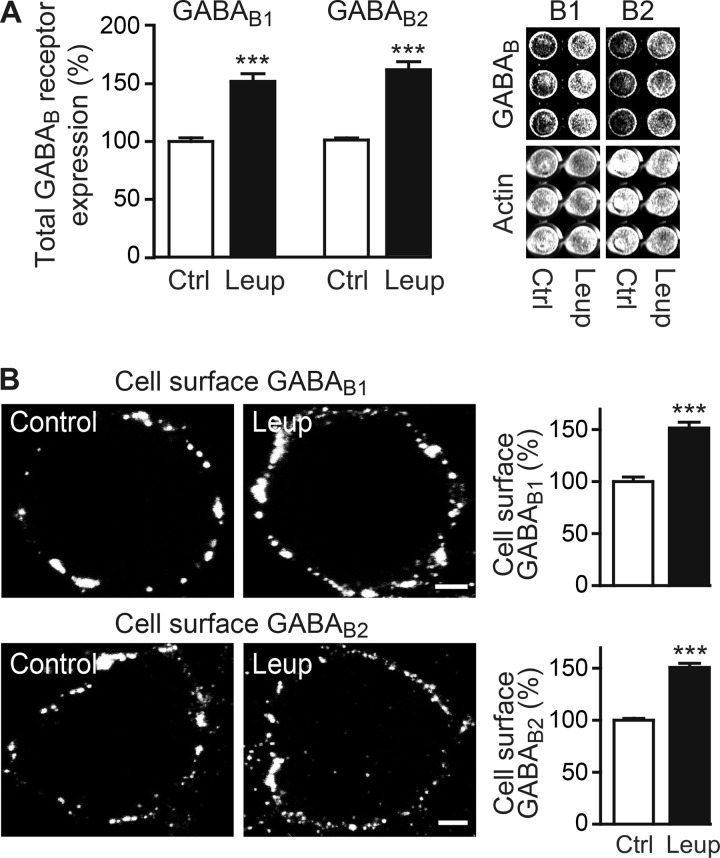
**Expression level of GABA_B_ receptors is controlled by lysosomes.** *A,* total expression level of GABA_B_ receptors is increased in neurons after blocking lysosomal activity. Cortical neurons were incubated for 12 h with 100 μm leupeptin (*Leup*) followed by immunostaining for total GABA_B1_ and GABA_B2_ as well as for actin using the in-cell Western technique. Neurons not treated with leupeptin served as controls (*Ctrl*). *Right,* representative images of an in-cell Western blot. *Left, graph* shows the quantification of fluorescence intensities normalized to the corresponding actin signals. Fluorescence intensities for GABA_B1_ and GABA_B2_ in control neurons were set to 100%. The data represent the mean ± S.E. of 30 cultures from three independent experiments. ***, *p* < 0.0001; two-tailed unpaired *t* test. *B,* expression of cell surface GABA_B_ receptors is increased in neurons after inhibiting lysosomal activity. Cortical neurons were treated as indicated in *A* and immunostained for cell surface GABA_B1_ and GABA_B2_. *Left,* representative images of the soma of stained neurons. *Scale bar,* 5 μm. *Right, graphs* show the quantification of fluorescence intensities. Fluorescence intensities for GABA_B1_ and GABA_B2_ in control neurons were set to 100%. The data represent the mean ± S.E. of 30–40 neurons from three independent experiments. ***, *p* < 0.0001; two-tailed unpaired *t* test.

GABA_B_ receptors undergo fast constitutive dynamin and clathrin-dependent endocytosis. Most of the receptors are recycled to the plasma membrane, although a minor fraction is sorted to the lysosomal degradation pathway (9–11, 20, 21). However, it is currently not known whether interfering with lysosomal degradation affects the expression of cell surface expression of GABA_B_ receptors. Blocking lysosomal degradation in cultured cortical neurons with leupeptin for 12 h considerably increased total (GABA_B1_, 151 ± 6%; GABA_B2_, 160 ± 7% of control; Fig. 1*A*) as well as cell surface expression of GABA_B_ receptors (GABA_B1_, 146 ± 9%; GABA_B2_, 147 ± 9% of control; Fig. 1*B*) to a similar extent. This suggests that constitutive lysosomal degradation is one factor determining the availability of GABA_B_ receptors at the cell surface for signaling.

##### Lys-63-linked Ubiquitination Is Involved in Lysosomal Degradation of GABA_B_ Receptors

**FIGURE 2.**
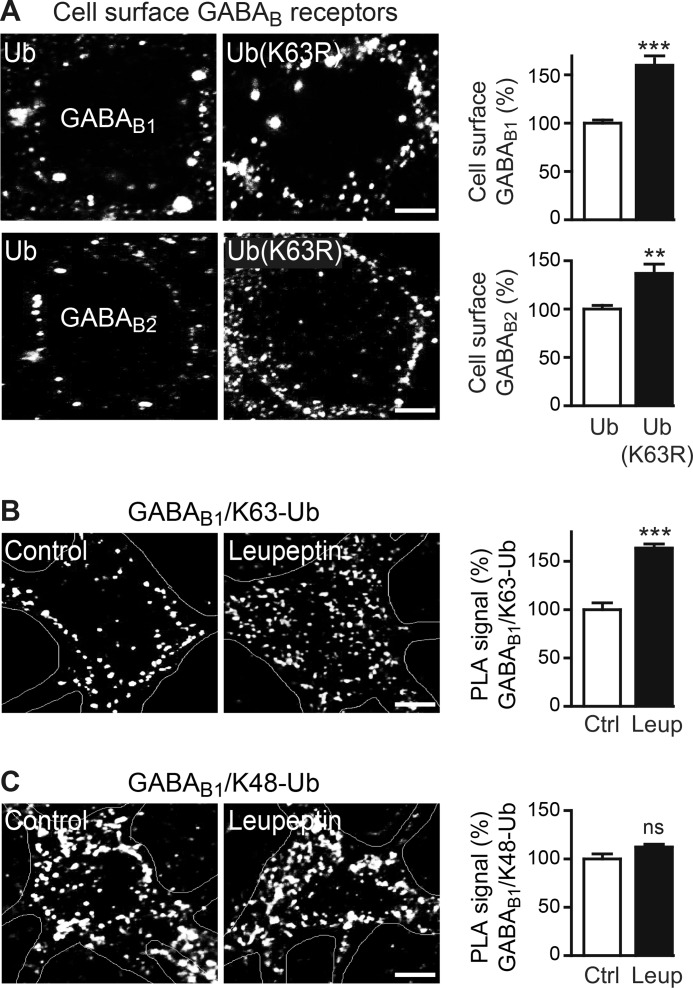
**Expression level of GABA_B_ receptors is regulated by Lys-63-linked ubiquitination.** *A*, interference with Lys-63-linked ubiquitination increased the expression level of cell surface GABA_B_ receptors. Neurons were transfected with wild-type ubiquitin (*Ub*) or a ubiquitin mutant unable to form Lys-63-linked chains (*Ub*(*K63R*)) and analyzed for GABA_B_ receptor expression using GABA_B1_ as well as GABA_B2_ antibodies. *Left,* representative images of stained neuronal somata (*scale bar,* 5 μm). *Right,* quantification of fluorescence intensities. The fluorescence signal of neurons transfected with wild-type ubiquitin was set to 100%. The data represent the mean ± S.E. of 30–34 neurons from three (GABA_B1_) and two (GABA_B2_) independent experiments. **, *p* < 0.004; ***, *p* < 0.0001; two-tailed unpaired *t* test. *B*, inhibition of lysosomal activity enhanced Lys-63-linked ubiquitination of GABA_B_ receptors. Cortical neurons were incubated for 12 h with or without (control) 100 μm leupeptin (*Leup*) and analyzed for Lys-63-linked ubiquitination by *in situ* PLA using antibodies directed against GABA_B1_ and Lys-63-linked ubiquitin (*white dots* in representative images, *scale bar,* 5 μm). *Right,* quantification of *in situ* PLA signals. The data represent the mean ± S.E. of 30–40 neurons from three independent experiments. ***, *p* < 0.00001; two-tailed unpaired *t* test. *Ctrl*, control. *C*, inhibition of lysosomal activity did not affect Lys-48-linked ubiquitination of GABA_B_ receptors. Cortical neurons were treated as in *B* and analyzed for Lys-48-linked ubiquitination by *in situ* PLA using antibodies directed against GABA_B1_ and Lys-48-linked ubiquitin (*white dots* in representative images, *scale bar,* 5 μm). *Right*, quantification of *in situ* PLA signals. The data represent the mean ± S.E. of 27–37 neurons from three independent experiments; *n.s.*, *p* > 0.05; two-tailed unpaired *t* test.

The signal that sorts GABA_B_ receptors to lysosomal degradation is unknown. Lys-48-linked ubiquitination tags proteins for degradation in proteasomes, whereas Lys-63-linked ubiquitination is involved in non-proteolytic functions and can serve as a sorting signal for lysosomal degradation (1). To test whether Lys-63-linked ubiquitination is involved in degrading GABA_B_ receptors, we transfected neurons with a mutant of ubiquitin that is not able to form Lys-63-linked chains (Ub(K63R)) and analyzed them for cell surface expression of GABA_B_ receptors. Inhibition of Lys-63-linked ubiquitination by overexpression of Ub(K63R) increased the expression level of cell surface GABA_B_ receptors (GABA_B1_, 162 ± 12%; GABA_B2_, 136 ± 9% of control neurons transfected with wild-type ubiquitin; Fig. 2*A*), suggesting that GABA_B_ receptor levels are regulated by Lys-63-linked ubiquitination.

Next we tested whether regulation of GABA_B_ receptor levels by lysosomal degradation requires direct Lys-63-linked ubiquitination of the receptor by *in situ* PLA using antibodies directed against GABA_B1_ and Lys-63-linked ubiquitin. Under basal conditions, GABA_B_ receptors exhibited Lys-63-linked ubiquitination, which considerably increased upon inhibition of lysosomal activity with leupeptin (164 ± 8% of control, Fig. 2*B*). In contrast, Lys-48-linked ubiquitination (which targets the receptors to proteasomal degradation (7, 8)) remained unaffected by blocking lysosomal activity (Fig. 2*C*). This suggests that direct Lys-63-linked ubiquitination of GABA_B_ receptors regulates lysosomal degradation of GABA_B_ receptors.

##### Identification of Lys-63-linked Ubiquitination Sites in GABA_B1_

**FIGURE 3.**
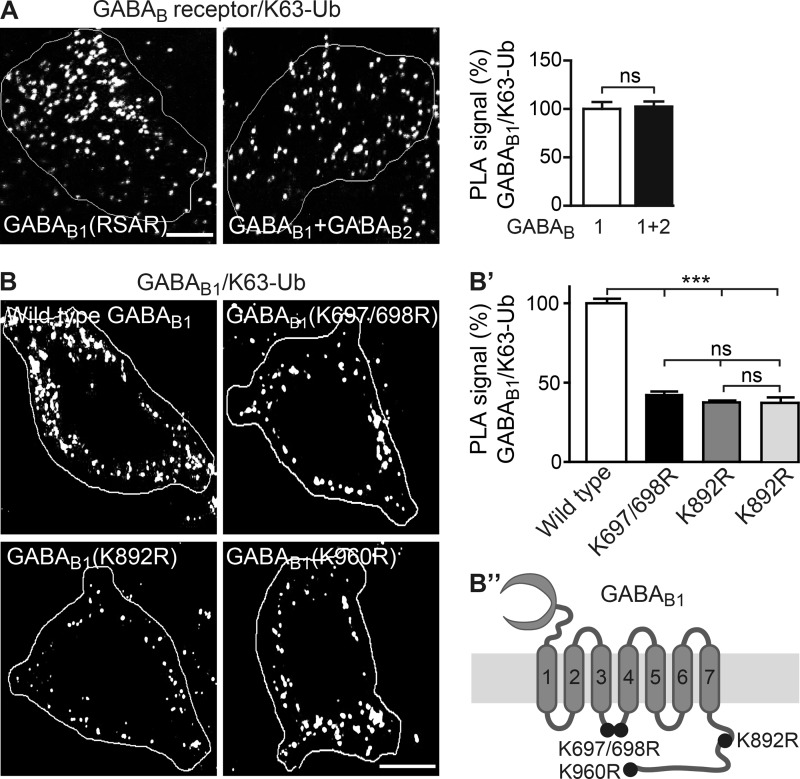
**Identification of Lys-63-linked ubiquitination sites in GABA_B1_.** *A*, GABA_B1_ is the main target for Lys-63-linked ubiquitination. HEK293 cells were either transfected with a GABA_B1_ mutant containing an inactivated ER retention signal (*GABA_B1a_*(*RSAR*)), which permits ER exit and cell surface targeting of the subunit when expressed alone, or with GABA_B1_ and GABA_B2_ and tested for Lys-63-linked ubiquitination by *in situ* PLA using GABA_B1_ antibodies in combination with an antibody detecting Lys-63-linked ubiquitin (*white dots* in representative images, *scale bar,* 7 μm). The data represent the mean ± S.E. of 47–49 neurons from three independent experiments. *ns*, *p* > 0.05; two-tailed unpaired *t* test. *B*, decreased Lys-63-linked ubiquitination of GABA_B1_(Lys → Arg) mutants. Cortical neurons were transfected with HA-tagged wild-type GABA_B1a_, HA-tagged GABA_B1a_(K697R/K698R), HA-tagged GABA_B1a_(K892R), or HA-tagged GABA_B1a_(K960R) together with wild-type GABA_B2_ and analyzed for Lys-63-linked ubiquitination by *in situ* PLA using antibodies directed against the HA tag and Lys-63-linked ubiquitin (*white dots* in representative images, *scale bar,* 7 μm). *B′,* quantification of *in situ* PLA signals. *B″,* schematic depicting the location of Lys → Arg mutations in GABA_B1_. The data represent the mean ± S.E. of 26–35 neurons from three independent experiments. *ns, p* > 0.05; ***, *p* < 0.0001; one-way ANOVA, Bonferroni's Multiple Comparison test.

For identification of Lys-63-linked ubiquitination sites in GABA_B_ receptors, we first determined whether GABA_B1_ or GABA_B2_ is the main target. HEK293 cells were either transfected with a GABA_B1_ mutant (GABA_B1a_(RSAR)) containing an inactivated ER retention signal, which permits ER exit and cell surface targeting of the subunit when expressed in the absence of GABA_B2_ (22), or with a combination of GABA_B1_ and GABA_B2_ and tested for Lys-63-linked ubiquitination with *in situ* PLA using antibodies directed against GABA_B1_ or GABA_B2_ and Lys-63-linked ubiquitin. We detected no difference in Lys-63-linked ubiquitination between HEK cells expressing GABA_B1_ alone and those expressing GABA_B1_ plus GABA_B2_, suggesting that GABA_B1_ is the main target for Lys-63-linked ubiquitination (Fig. 3*A*).

We then searched for potential lysine residues serving as ubiquitination sites in the GABA_B1_ sequence by an *in silico* analysis. Four lysines with a high probability of being ubiquitinated were identified as follows: two in the cytoplasmic loop linking transmembrane domains three and four and two in the C-terminal domain (Fig. 3*B″*). Inactivation of these sites by mutation to arginine (Lys → Arg) yielded the three mutants GABA_B1a_(K697R/K698R), GABA_B1a_(K892R), and GABA_B1a_(K960R). To test whether these sites are ubiquitinated, HEK293 cells were transfected with either wild-type GABA_B1a_ or one of the GABA_B1a_(Lys → Arg) mutants along with GABA_B2_ and analyzed for Lys-63-linked ubiquitination by *in situ* PLA. Numerous *in situ* PLA signals in cells transfected with wild-type GABA_B1a_ indicated that a fraction of GABA_B1a_ is Lys-63-linked ubiquitinated under basal conditions. In contrast, all three mutant GABA_B1a_ displayed strongly reduced Lys-63-linked ubiquitination (GABA_B1a_(K697R/K698R), 43 ± 3%; GABA_B1a_(K892R), 38 ± 3%; GABA_B1a_(K960R), 37 ± 3%, of wild-type GABA_B1a_; Fig. 3*B*). This result indicates that lysines 697 and/or 698 and lysine 982 and lysine 960 in GABA_B1_ can be Lys-63-linked ubiquitinated under basal conditions.

##### Ubiquitination of GABA_B1_ Regulates Cell Surface Expression of GABA_B_ Receptors

**FIGURE 4.**
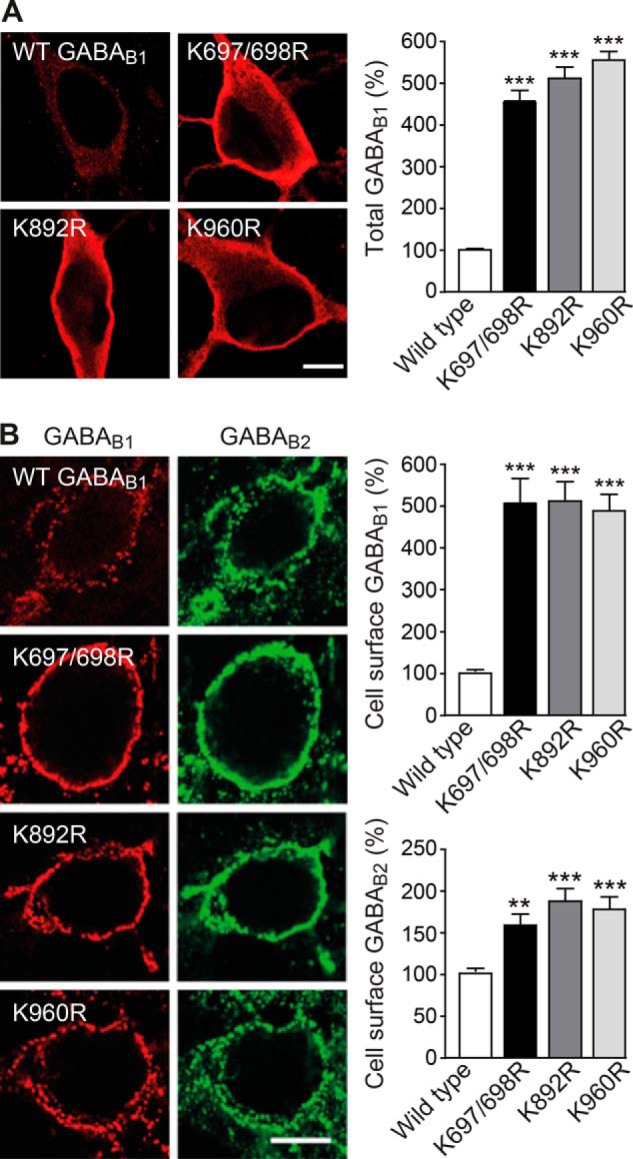
**GABA_B1_(Lys → Arg) mutants exhibit increased total and cell surface expression.** *A*, increased total expression levels of GABA_B1a_(Lys → Arg) mutants. Neurons were transfected with HA-tagged wild-type GABA_B1a_, HA-tagged GABA_B1a_(K697R/K698R), HA-tagged GABA_B1a_(K892R), or HA-tagged GABA_B1a_(K960R) together with wild-type GABA_B2_ and analyzed for the expression level of transfected GABA_B1_ using antibodies directed against the HA tag. *Left,* representative images (*scale bar,* 7 μm). *Right,* quantification of fluorescence signals. The fluorescence signals of neurons transfected with wild-type GABA_B1_ were set to 100%. The data represent the mean ± S.E. of 23–27 neurons per experimental condition derived from three independent experiments. ***, *p* < 0.0001; one-way ANOVA, Dunnett's Multiple Comparison test. *B*, increased cell surface expression levels of GABA_B1a_(Lys → Arg) mutants. Neurons were transfected with HA-tagged wild-type GABA_B1a_, HA-tagged GABA_B1a_(K697R/K698R), HA-tagged GABA_B1a_(K892R), or HA-tagged GABA_B1a_(K960R) together with wild-type GABA_B2_ and analyzed for cell surface expression levels of transfected GABA_B1_ as well as transfected plus endogenous GABA_B2_ using antibodies directed against the HA tag and GABA_B2_, respectively. *Left,* representative images (*scale bar,* 7 μm). *Right,* quantification of fluorescence signals. The fluorescence signals of neurons transfected with wild-type GABA_B1a_ or wild-type GABA_B2_, respectively, was set to 100%. The data represent the mean ± S.E. of 26–28 neurons per experimental condition derived from three independent experiments. **, *p* < 0.001; ***, *p* < 0.0001; one-way ANOVA, Dunnett's Multiple Comparison test.

To analyze the effect of Lys-63-linked ubiquitination on cell surface expression of GABA_B_ receptors, we transfected neurons with wild-type GABA_B1a_ or GABA_B1a_(Lys → Arg) mutants along with GABA_B2_ and immunostained for their total and cell surface expression levels. Total as well as cell surface expression of all three GABA_B1a_ mutants was considerably increased as compared with transfected wild-type GABA_B1a_ (total, GABA_B1a_(K697R/K698R), 457 ± 26%; GABA_B1a_(K892R), 511 ± 30%; and GABA_B1a_(K960R), 551 ± 22%, of wild-type GABA_B1a_; cell surface, GABA_B1a_(K697R/K698R), 508 ± 52%; GABA_B1a_(K892R), 504 ± 48%; and GABA_B1a_(K960R), 482.2 ± 42% of wild-type GABA_B1_; Fig. 4, *A* and *B*). Likewise, the cell surface expression of GABA_B2_ in neurons transfected with GABA_B1a_(Lys → Arg) mutants was significantly increased (GABA_B2_ in GABA_B1a_ (K697R/K698R)-transfected neurons, 158 ± 14%; GABA_B2_ in GABA_B1a_(K892R)-transfected neurons, 187 ± 17%; GABA_B2_ in GABA_B1a_(K960R)-transfected neurons, 178 ± 16% of control; Fig. 4*B*). The considerably lower increase in GABA_B2_ cell surface expression as compared with mutant GABA_B1a_ was due to the fact that in the case of GABA_B1_ only transfected subunits were assayed (HA-tagged), but in the case of GABA_B2_ transfected as well as endogenously expressed subunits were detected. The results demonstrate that inactivation of any of the ubiquitination sites in GABA_B1_ (Lys-697/698, Lys-892, and Lys-960) decreased or prevented degradation of GABA_B_ receptors and therefore increased their cell surface expression.

##### Lysosomal Targeting of GABA_B_ Receptors Is Regulated by Ubiquitination of GABA_B1_

**FIGURE 5.**
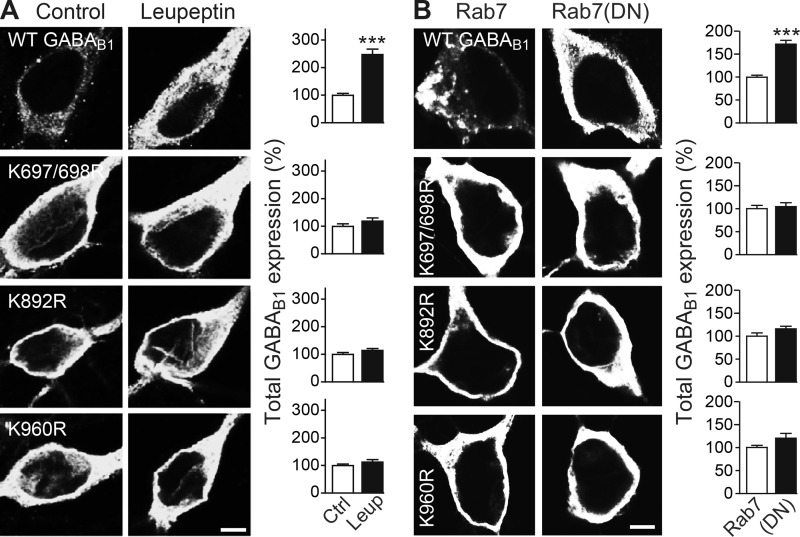
**Expression levels of GABA_B1a_(Lys → Arg) mutants are unaffected by inhibition of lysosomal degradation.** *A*, total expression levels of GABA_B1a_(Lys → Arg) mutants are unaffected by blocking lysosomal activity with leupeptin. Neurons were transfected with HA-tagged wild-type GABA_B1a_ or HA-tagged GABA_B1a_(Lys → Arg) mutants together with GABA_B2_, incubated with 100 μm leupeptin for 12 h, followed by immunostaining for transfected HA-tagged GABA_B1_ using HA antibodies. *Left,* representative images of untreated neurons (*control, left*) and of neurons incubated with leupeptin (*right, scale bar,* 7 μm). *Right,* quantification of fluorescence intensities. The fluorescence intensity of GABA_B1a_ from untreated neurons (control) was set to 100%. The data represent the mean ± S.E. of 27–34 neurons per experimental condition derived from three independent experiments. ***, *p* < 0.0001, two-tailed unpaired *t* test. *B*, total expression levels of GABA_B1a_(Lys → Arg) mutants are unaffected upon blocking lysosomal targeting by inactivation of Rab7. Neurons were transfected with HA-tagged wild-type GABA_B1a_ or GABA_B1a_(Lys → Arg) mutants together with GABA_B2_ and with either wild-type Rab7 or with a non-functional mutant of Rab7 (Rab7(DN)) and analyzed for total expression levels of transfected GABA_B1_ using HA antibodies. *Left,* representative images depicting total expression of transfected GABA_B1a_ (*scale bar,* 7 μm). *Right,* quantification of fluorescence intensities. The fluorescence intensities of GABA_B1a_ coexpressed with wild-type Rab7 were set to 100%. The data represent the mean ± S.E. of 27–34 neurons derived from three independent experiments. ***, *p* < 0.0001, two-tailed unpaired *t* test.

The increased total and cell surface expression levels of GABA_B1a_(Lys → Arg) mutants and their reduced Lys-63-linked ubiquitination suggest that ubiquitination of these lysine residues serves as signals for sorting the receptors to lysosomes for degradation. If this is the case, GABA_B1a_(Lys → Arg) mutants should be resistant to lysosomal degradation, and their expression levels should not increase upon blocking lysosomal degradation. Indeed, in contrast to the expression level of wild-type GABA_B1a_, those of all three GABA_B1a_(Lys → Arg) mutants remained unaffected by inhibition of lysosomal degradation with leupeptin (wild-type GABA_B1a_, 249 ± 30%; GABA_B1a_ (K697R/K698R), 111 ± 5%; GABA_B1a_ (K892R), 109 ± 5%; and GABA_B1a_ (K960R), 108 ± 4% of control; Fig. 5*A*).

To confirm this finding, we prevented lysosomal degradation by overexpressing a functionally inactive mutant of the small GTPase Rab7 (Rab7(DN)). Rab7 mediates trafficking from early endosomes via late endosomes to lysosomes (23), and therefore overexpression of Rab7(DN) disrupts this pathway. In line with the pharmacological data, overexpression of Rab7(DN) considerably enhanced total expression of wild-type GABA_B1a_ but did not significantly affect the expression levels of GABA_B1a_(Lys → Arg) mutants (wild-type GABA_B1a_, 174 ± 11%; GABA_B1a_ (K697R/K698R), 105 ± 8%; GABA_B1a_ (K892R), 117 ± 8%; and GABA_B1a_ (K960R), 126 ± 9% of control; Fig. 5*B*). This indicates that preventing ubiquitination of specific sites in GABA_B1_ excluded the mutant receptors from entering the endosomal pathway that directs proteins to the lysosome. Therefore, our observations suggest that ubiquitination of multiple lysine residues in GABA_B1_ receptors regulates lysosomal degradation of GABA_B_ receptors.

##### E3 Ligase Mindbomb-2 (MIB2) Mediates Lys-63-linked Ubiquitination of GABA_B1_

**FIGURE 6.**
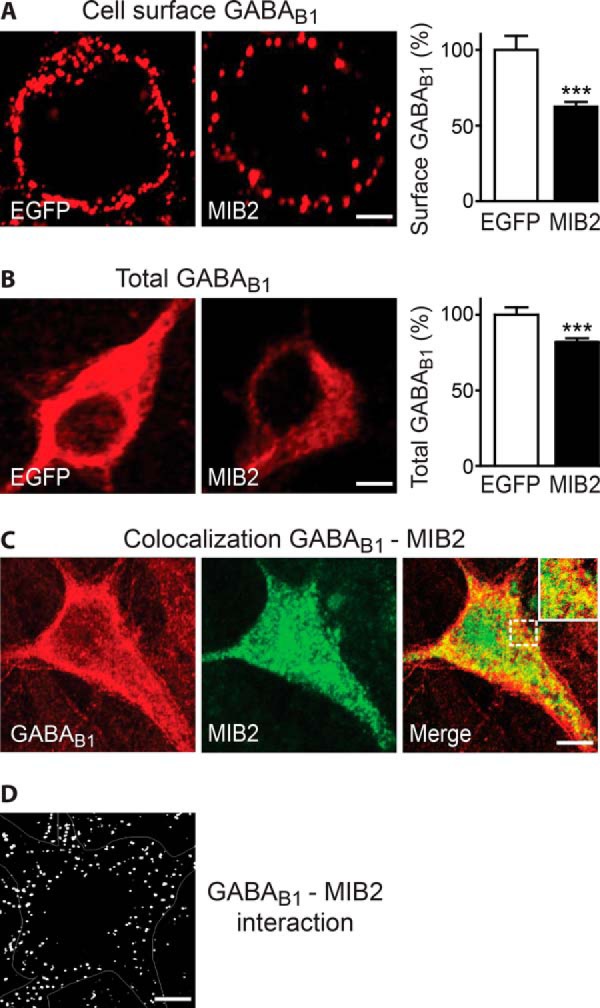
**E3 ligase MIB2 colocalizes with GABA_B_ receptors and affects their expression level.** *A* and *B*, overexpression of MIB2 in neurons reduced expression levels of GABA_B_ receptors. Neurons were either transfected with EGFP (controls) or MIB2 and analyzed for cell surface (*A*) or total (*B*) expression of GABA_B_ receptors. *Left,* representative images (*scale bar,* 5 μm). *Right,* quantification of fluorescence intensities. The data represent the mean ± S.E. of 30 (*A*) and 45 (*B*) neurons derived from two independent experiments. ***, *p* < 0.0001, two-tailed unpaired *t* test. *C*, MIB2 and GABA_B_ receptors extensively colocalize in cortical neurons. Neurons were simultaneously stained for GABA_B1_ (*red*) and MIB2 (*green*). *Scale bar,* 5 μm. *D,* GABA_B_ receptors interact with MIB2. Neurons were stained for GABA_B1_ and MIB2 and analyzed for interaction via *in situ* PLA. *Scale bar,* 5 μm.

In the next step, we aimed at identifying the E3 ligase mediating Lys-63-linked ubiquitination of GABA_B_ receptors. A recent comprehensive proteomic study determined proteins that robustly interact with GABA_B_ receptors and most likely build basic GABA_B_ receptor signaling complexes (24). A few E3 ligases, which did not pass their stringent criteria for a robustly associated protein and thus were not regarded as a permanent member of a basic GABA_B_ receptor signaling complex, emerged in their screens (MIB2, TRIM9, and MYCBP2; additional tested E3 ligases were RNF112, RNF144, RNF167, and RNF152). Upon overexpression of those E3 ligases in neurons, we found that MIB2 significantly reduced cell surface (59 ± 3% of control, Fig. 6*A*) as well as total (78 ± 3% of control, Fig. 6*B*) GABA_B_ receptor expression. MIB2 extensively colocalized with GABA_B_ receptors in neurons (Fig. 6*C*) and interacted with GABA_B_ receptors as tested by *in situ* PLA (Fig. 6*D*).

**FIGURE 7.**
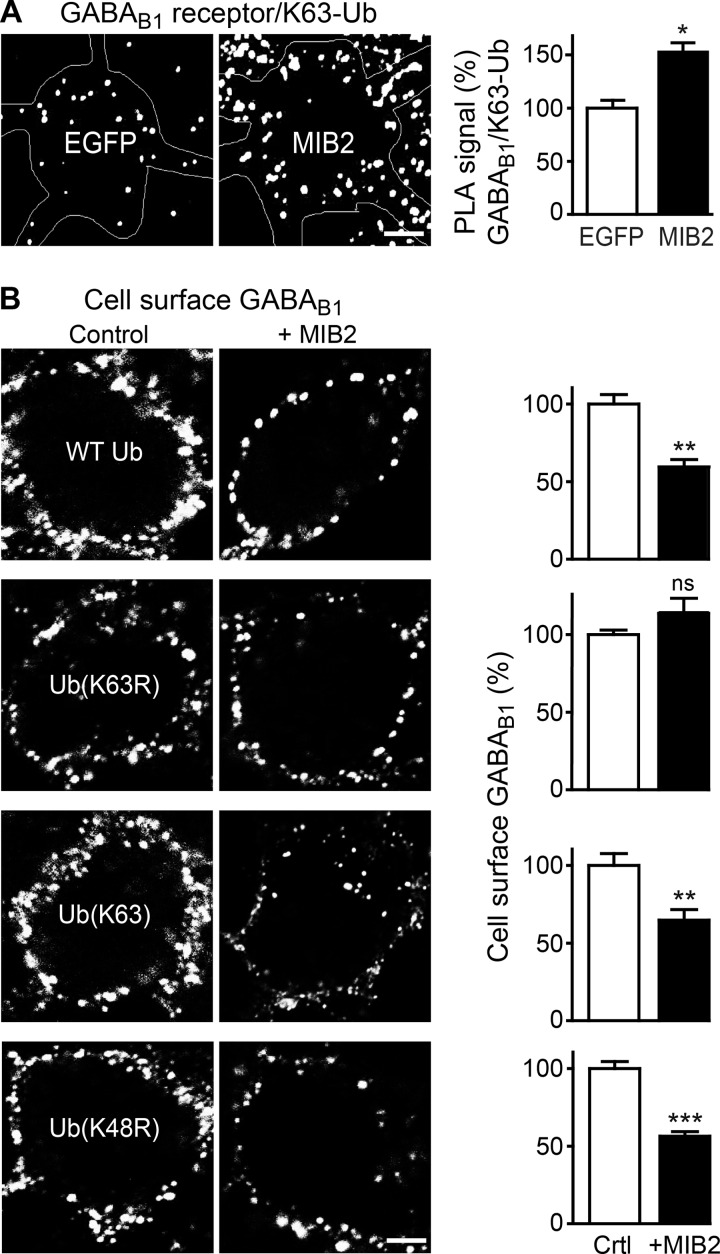
**MIB2-induced down-regulation of GABA_B_ receptors is mediated by Lys-63-linked ubiquitination.** *A*, MIB2 mediates Lys-63-linked ubiquitination of GABA_B_ receptors. Neurons were transfected with EGFP (*control*) or MIB2 and tested for Lys-63-linked ubiquitination of GABA_B_ receptors using *in situ* PLA using antibodies directed against GABA_B1_ and Lys-63-linked ubiquitin (*white dots* in representative images, *scale bar,* 5 μm). The *graph* depicts quantification of the *in situ* PLA signals. The data represent the mean ± S.E. of 20 neurons per condition derived from two independent experiments. *, *p* < 0.05, two-tailed unpaired *t* test. *B*, MIB2-induced down-regulation of GABA_B_ receptors is mediated by Lys-63-linked ubiquitination. Neurons were transfected with either wild-type ubiquitin (*WT Ub*), mutant ubiquitin that cannot form Lys-63 linkages (*Ub*(*K63R*), mutant ubiquitin that can only form Lys-63 linkages (*Ub*(*Lys-63*), and mutant ubiquitin that is deficient in forming Lys-48 linkages (*Ub*(*K48R*)), and with or without (*control*) MIB2 followed by determination of cell surface GABA_B_ receptors using GABA_B1_ antibodies. *Left,* representative images (*scale bar,* 5 μm). *Right,* quantification of fluorescence intensities. The data represent the mean ± S.E. of 20–22 neurons for each condition derived from two independent experiments. *ns, p* > 0.05; **, *p* < 0.005; ***, *p* < 0.0005, two-tailed unpaired *t* test; *ns, p* > 0.05.

Next we analyzed whether MIB2 mediates Lys-63-linked ubiquitination of GABA_B_ receptors. To demonstrate directly Lys-63-linked ubiquitination of GABA_B_ receptors by MIB2, we overexpressed MIB2 in neurons and tested for increased Lys-63-linked ubiquitination using *in situ* PLA. In fact, overexpression of MIB2 in neurons increased Lys-63-linked ubiquitination of GABA_B_ receptors to 156 ± 16% of controls (Fig. 7*A*). This result was corroborated by the observation that overexpression of mutant ubiquitin, which cannot form Lys-63 linkages (Ub(K63R)), inhibited the MIB2 effect on GABA_B_ receptors (Fig. 7*B*). In contrast overexpression of MIB2 in neurons with either wild-type ubiquitin (WT Ub (59 ± 8% of control)), mutant ubiquitin that can only form Lys-63 linkages (Ub(Lys-63), 64 ± 6% of control), or mutant ubiquitin that is deficient in forming Lys-48 linkages (Ub(K48R), 57 ± 6% of control) did not affect MIB2-mediated down-regulation of GABA_B_ receptors (Fig. 7*B*).

**FIGURE 8.**
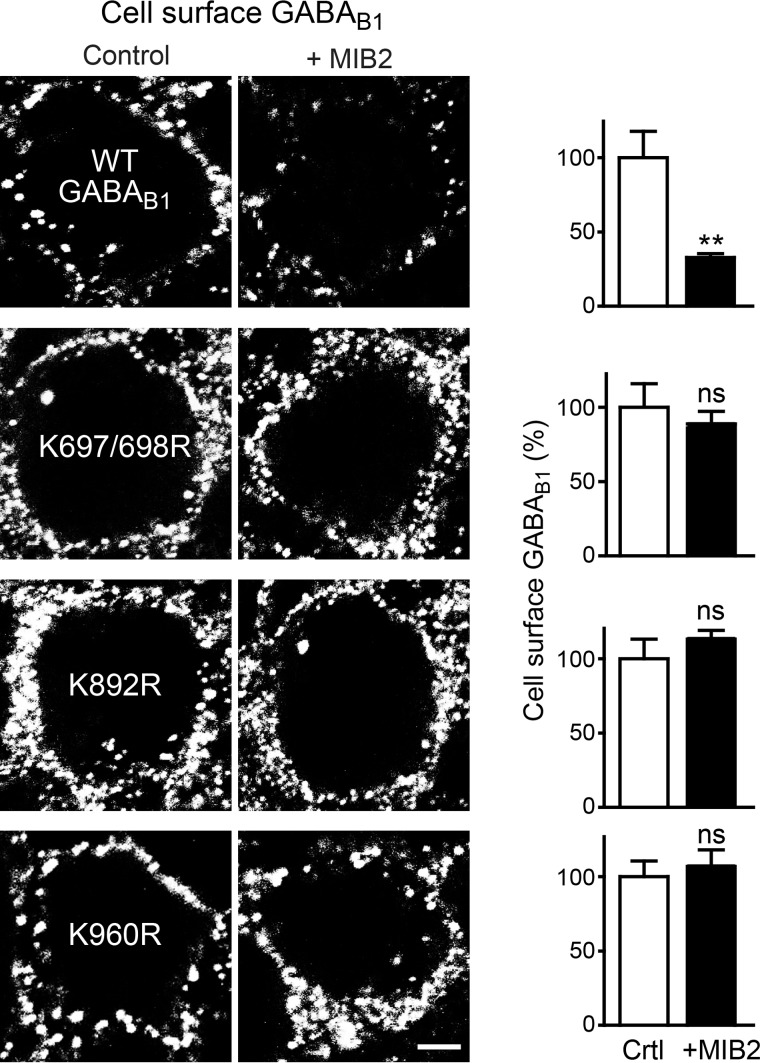
**MIB2 mediates Lys-63-linked ubiquitination of GABA_B_ receptors.** GABA_B1a_(Lys → Arg) mutants, which are partially resistant to Lys-63-linked ubiquitination, are not affected by overexpression of MIB2. Neurons were transfected with HA-tagged wild-type GABA_B1a_ (*WT GABA_B1_*), GABA_B1a_(*K697/698R*), GABA_B1a_(*K892R*) or GABA_B1a_(*K960R*) with (+MIB2) or without (control) MIB2 and analyzed for cell surface expression of wild-type and mutant GABA_B1a_ using HA antibodies. *Left,* representative images, *scale bar,* 5 μm. *Right,* quantification of fluorescence intensities. The data represent the mean ± S.E. of 19–24 neurons per experimental condition derived from two independent experiments. *ns, p* > 0.05; **, *p* < 0.005, two-tailed unpaired *t* test.

To further substantiate that Lys-63-linked ubiquitination is mediated via MIB2, we analyzed the effect of overexpression of MIB2 on the three GABA_B1a_(Lys → Arg) mutants, which are partially resistant to Lys-63-linked ubiquitination. In this set of experiments, overexpression of MIB2 reduced cell surface expression of wild-type GABA_B1_ to 33 ± 4% of controls (Fig. 8). However, cell surface expression of all three mutants remained unaffected by overexpression of MIB2 (Fig. 8).

##### Sustained Activation of Glutamate Receptors Increases Lys-63-linked Ubiquitination of GABA_B_ Receptors via MIB2

**FIGURE 9.**
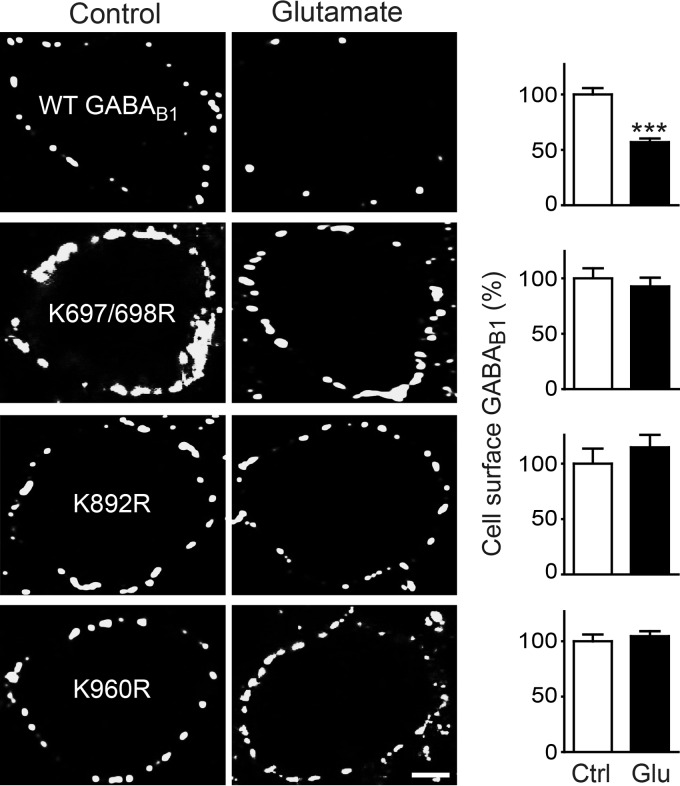
**GABA_B1a_(Lys → Arg) mutants are resistant to glutamate-induced down-regulation.** Neurons transfected with HA-tagged wild-type GABA_B1a_ or HA-tagged GABA_B1a_(Lys → Arg) mutants along with GABA_B2_ were incubated in the presence (glutamate) or absence (*control*) of 50 μm glutamate for 90 min followed by cell surface staining for transfected GABA_B1a_ using HA antibodies. *Left,* representative images, *scale bar,* 5 μm. *Right,* quantification of fluorescence intensities. The fluorescence intensity of neurons not treated with glutamate was set to 100%. The data represent the mean ± S.E. of 20–25 neurons per experimental condition derived from two independent experiments. ***, *p* < 0.0005, two-tailed unpaired *t* test.

Prolonged activation of glutamate receptors (AMPA as well as NMDA receptors) leads to down-regulation of GABA_B_ receptors via lysosomal degradation (16–18). To investigate whether Lys-63-linked ubiquitination of GABA_B_ receptors serves as a lysosomal sorting signal in this process, we first tested whether the three GABA_B1a_(Lys → Arg) mutants, which are partially resistant to Lys-63-linked ubiquitination, are resistant to glutamate-induced down-regulation. In contrast to the cell surface expression of wild-type GABA_B1a_ (56 ± 8% of control, Fig. 9), the levels of all three GABA_B1a_(Lys → Arg) mutants remained unaffected by glutamate (GABA_B1a_(K697R/K698R), 90 ± 12%; GABA_B1a_(K892R), 115 ± 11%; GABA_B1a_ (K960R), 108 ± 5% of control; Fig. 9). This suggests that Lys-63-linked ubiquitination of GABA_B1_ is the signal for down-regulating the receptors.

**FIGURE 10.**
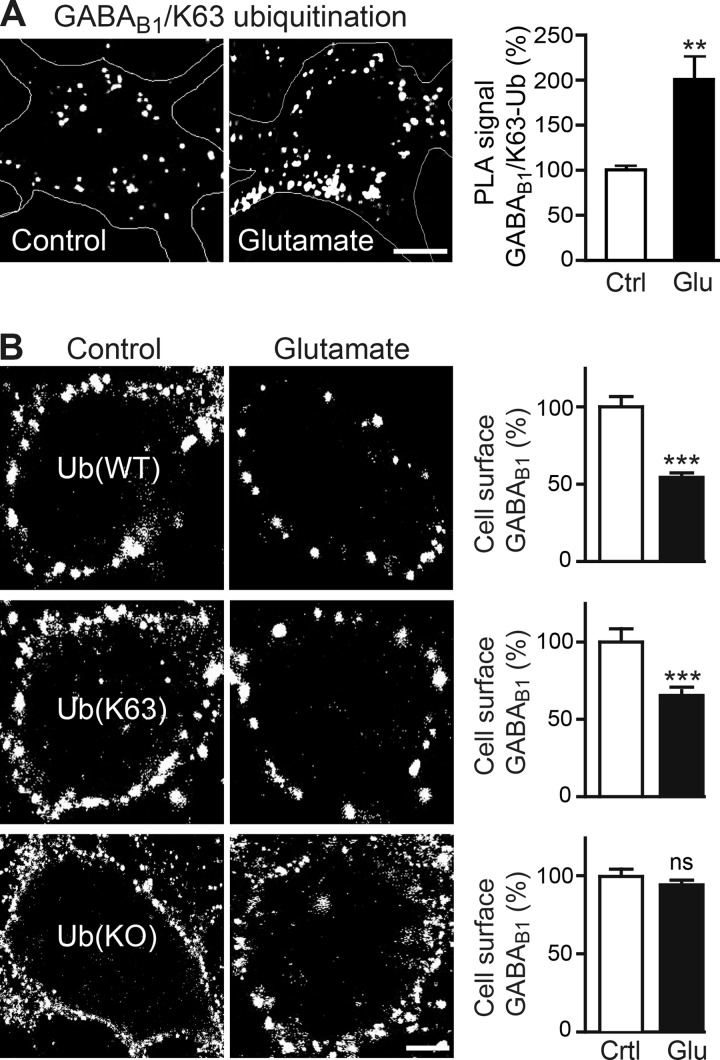
**Glutamate-induced down-regulation of GABA_B_ receptors is mediated by Lys-63-linked ubiquitination.** *A*, sustained activation of glutamate receptors enhanced Lys-63-linked ubiquitination of GABA_B_ receptors. Neurons were incubated for 60 min in the absence (*control*) or presence of 50 μm glutamate and analyzed for Lys-63-linked ubiquitination by *in situ* PLA using antibodies directed against GABA_B1_ and Lys-63-linked ubiquitin (*white dots* in representative images, *scale bar,* 5 μm). The *graph* depicts quantification of the *in situ* PLA signals. The data represent the mean ± S.E. of 20 neurons derived from two independent experiments. **, *p* < 0.01; one-way ANOVA, Dunnett's Multiple Comparison test. *B*, preventing Lys-63-linked ubiquitination rendered GABA_B_ receptors resistant to glutamate-induced down-regulation. Neurons were transfected with wild-type ubiquitin (*Ub*(*WT*)), and mutants of ubiquitin that either permits only Lys-63-linked ubiquitination (*Ub*(*Lys-63*)) or prevents any kind of ubiquitin chain generation (*Ub*(*KO*)). Neurons were incubated for 90 min in the absence (*control*) or presence of 50 μm glutamate followed by determination of cell surface GABA_B_ receptors using GABA_B1_ antibodies. *Left,* representative images, *scale bar,* 5 μm. *Right,* quantification of fluorescence intensities. The data represent the mean ± S.E. of 30–36 neurons from three independent experiments. ***, *p* < 0.0001; two-tailed unpaired *t* test; *ns, p* > 0.05.

To directly test for ubiquitination of the receptors in this mechanism, we exposed cortical neurons for 30 min to glutamate and determined Lys-63-linked ubiquitination of the receptors via *in situ* PLA. As expected, sustained activation of glutamate receptors strongly increased Lys-63-linked ubiquitination of GABA_B_ receptors (203 ± 34% of control; Fig. 10*A*).

Next we tested whether preventing Lys-63-linked ubiquitination inhibits the down-regulation of GABA_B_ receptors after treating neurons with glutamate. For this, cortical neurons were transfected either with wild-type Ub, a mutant of ubiquitin in which all lysines were mutated to arginines thereby preventing chain elongation and thus any kind of polyubiquitination (Ub(KO)), or with a mutant in which all lysines were mutated to arginines except for Lys-63 (Ub(Lys-63), able to form only Lys-63-linked ubiquitination) and stained for cell surface GABA_B_ receptors after sustained glutamate application. Glutamate induced down-regulation of GABA_B_ receptors from the plasma membrane in neurons expressing wild-type ubiquitin (Ub(WT), 53 ± 5% of control; Fig. 10*B*) or the mutant that only permits Lys-63-linked ubiquitination (Ub(Lys-63), 61 ± 6% of control, Fig. 10*B*) but not in neurons expressing the mutant unable to build polyubiquitin chains (Ub(KO), 95 ± 11% of control, Fig. 10*B*).

**FIGURE 11.**
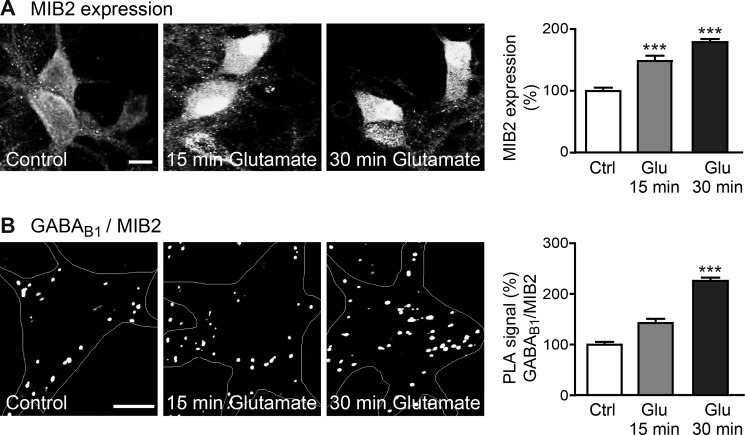
**Glutamate exposure increases the expression level of MIB2 and the MIB2-GABA_B_ receptor interaction.** *A*, increased MIB2 expression after glutamate exposure. Neurons were treated either for 15 or 30 min with glutamate and analyzed for MIB2 expression. *Left,* representative images, *scale bar,* 10 μm. *Right,* quantification of fluorescence intensities. The data represent the mean ± S.E. of 30 neurons from two independent experiments. ***, *p* < 0.0001; one-way ANOVA, Dunnett's Multiple Comparison test. *B*, increased interaction of GABA_B_ receptors with MIB2 after glutamate exposure. Neurons were treated either for 15 or 30 min with glutamate and analyzed for the interaction of MIB2 with GABA_B_ receptors using *in situ* PLA. *Left,* representative images, *scale bar,* 5 μm. *Right,* quantification of the *in situ* PLA signals. The data represent the mean ± S.E. of 14 neurons from two independent experiments. ***, *p* < 0.0003; one-way ANOVA, Dunnett's Multiple Comparison test.

Finally, we analyzed whether MIB2 is involved in glutamate-induced down-regulation of the receptors. Interestingly, treatment of neurons with glutamate significantly increased MIB2 expression in neurons (15 min of glutamate, 150 ± 9% of control; 30 min of glutamate, 179 ± 8% of control; Fig. 11*A*) and strongly increased the interaction of MIB2 with GABA_B_ receptors as tested with *in situ* PLA (15 min of glutamate, 155 ± 13% of control; 30 min of glutamate, 218 ± 25% of control; Fig. 11*B*).

These findings suggest that sustained activation of glutamate receptors induces MIB2-mediated Lys-63-linked ubiquitination of GABA_B_ receptors, promoting their lysosomal degradation.

## Discussion

The signaling strength of G protein-coupled receptors largely depends on the number of receptors present in the plasma membrane. The mechanisms determining cell surface expression of the receptors include exocytosis, endocytosis, recycling, and degradation. GABA_B_ receptors assemble into heterodimeric GABA_B1,2_ complexes in the ER, which is a prerequisite for their ER exit and forward trafficking to the plasma membrane. After reaching the cell surface, GABA_B_ receptors are constitutively internalized and either recycled to the plasma membrane or degraded in lysosomes (25). Both forward trafficking of GABA_B_ receptors to the cell surface as well as their residence time at the cell surface are tightly regulated by controlled degradation of the receptors. The amount of GABA_B_ receptors available for forward trafficking to the plasma membrane in the ER is adjusted by proteasomal degradation of the receptors via the ERAD machinery depending on the activity level of the neuron (7, 8). In contrast, the amount of receptors degraded in lysosomes after internalization from the cell surface depends on mechanisms sorting the endocytosed receptors to either lysosomes or recycling endosomes. Interfering with recycling rapidly depletes the receptors from the cell surface by redirecting them to the lysosomal degradation pathway (10). Rapid down-regulation of cell surface GABA_B_ receptors by rerouting the receptors to lysosomes appears to be associated with pathological conditions as it is induced by sustained activation of glutamate receptors, which is a characteristic of brain ischemia (16–19). The factors triggering lysosomal degradation of GABA_B_ receptors were unknown, however. The results of this study provide evidence that MIB2-mediated Lys-63-linked ubiquitination of GABA_B1_ sorts GABA_B_ receptors to lysosomes for degradation under physiological and pathological conditions.

We found that pharmacological inhibition of lysosomal activity increased not only total GABA_B_ receptor levels, which was expected due to the intracellular accumulation of the receptors (9), but also considerably enhanced cell surface expression of the receptors. This finding implies that regulating lysosomal degradation of GABA_B_ receptors directly affects their cell surface expression, which in turn determines the strength of GABA_B_ receptor signaling (7). Here we provide evidence that Lys-63-linked ubiquitination is required for lysosomal degradation of GABA_B_ receptors. First, blocking global Lys-63-linked ubiquitination by overexpressing a ubiquitin mutant (K63R) that is unable to form Lys-63-linked chains significantly increased cell surface expression of GABA_B_ receptors. Second, blocking lysosomal activity considerably increased the level of Lys-63-linked ubiquitination of GABA_B_ receptors while leaving the level of Lys-48-linked ubiquitination, which tags the receptors for proteasomal degradation (7), unaffected. Mutational inactivation of potential ubiquitination sites in GABA_B1_ (Lys-697/Lys-698, Lys-892, and Lys-960) strongly decreased Lys-63-linked ubiquitination of GABA_B_ receptors containing the respective GABA_B1_ mutant and prevented their lysosomal degradation as indicated by their dramatically increased expression level and insensitivity to the effect of blocking lysosomal degradation (either by inhibiting lysosomal proteases by leupeptin or by overexpression of a functionally inactive mutant of Rab7, which inhibits transport of cargo from late endosomes to the lysosome and blocks lysosome biogenesis). Any of the three GABA_B1_ mutants (K697R/K698R, K892R, and K960R) appeared to completely prevent lysosomal degradation of the receptors, suggesting that ubiquitination of Lys-697/Lys-698, Lys-892, and Lys-960 in GABA_B1_ is mandatory for lysosomal degradation of GABA_B_ receptors. A similar situation was reported for targeting EGF receptors to lysosomal degradation. Multiple Lys-63-linked ubiquitination sites were identified, and mutation of each site prevented degradation of the receptors (26). It is currently unclear at which stage of intracellular sorting Lys-697/Lys-698, Lys-892, and Lys-960 in GABA_B1_ need to be ubiquitinated. They may be ubiquitinated simultaneously at a certain sorting step, or alternatively, they may be sequentially ubiquitinated at distinct sorting checkpoints. Addressing this issue in relation to the ESCRT pathway for sorting the receptors to lysosomes is an important question that requires further investigation.

Lysosomal degradation of G protein-coupled receptors is predominantly mediated via the ESCRT machinery (27), which guides mono- and Lys-63-linked ubiquitinated membrane proteins to lysosomes (28). Therefore, our observation that Lys-63-linked ubiquitination tags GABA_B_ receptors for lysosomal degradation indicates that the ESCRT machinery also sorts GABA_B_ receptors to lysosomes. This view is supported by the finding that the ESCRT I complex component TGS101 (29) is required for lysosomal degradation of GABA_B_ receptors (13). In addition, the deubiquitination enzyme USP14 has been implicated in lysosomal degradation of GABA_B_ receptors (15). Deubiquitination of proteins is an integral part of ESCRT-mediated degradation. Deubiquitinases associated with the ESCRT-0 complex are thought to rescue proteins from degradation by deubiquitination at an early step of lysosomal targeting, whereas deubiquitinases recruited to ESCRT-III recycle ubiquitin before the cargo protein is being degraded in the lysosome (14). However, USP14 appears not to be involved in these classical functions. Instead, USP14 interacts with GABA_B_ receptors and contributes to their lysosomal targeting independent of its deubiquitinating activity, in an as yet undefined way (15).

So far, information on the E3 ubiquitin ligases mediating ubiquitination of GABA_B_ receptors is almost entirely lacking. We previously found that the prototypical ERAD E3 ligase Hrd1 interacts with GABA_B_ receptors residing in the ER and is most likely responsible for Lys-48-linked ubiquitination of GABA_B2_, which tags the receptors for proteasomal degradation (7). Here we identified MIB2 as the E3 ubiquitin ligase mediating Lys-63-linked ubiquitination of GABA_B1_, tagging the receptors for lysosomal degradation. MIB2 was detected in a proteomic screen, but it did not fulfill the rigorous criteria of the authors for a robustly GABA_B_ receptor-associated protein (24). However, we found that MIB2 in fact colocalized with GABA_B_ receptors in neurons and interacted with GABA_B_ receptor complexes as tested by *in situ* PLA.

MIB2 belongs to the class of RING (really interesting new gene) domain E3 ligases composed of two separate substrate recognition domains in its N-terminal portion and two RING domains with the ubiquitin ligase activity in the C-terminal portion (30). The best described function of MIB2 is the ubiquitination and internalization of Notch ligands (31). Because Notch signaling in the adult brain is involved in synaptic plasticity, memory, and learning, MIB2-deficient mice displayed impaired hippocampal long-term potentiation and spatial memory as well as contextual fear memory (32). Apart from regulating Notch signaling, MIB2 has been shown to control diverse systems. For instance, it mediates Lys-63-linked ubiquitination of TANK-binding kinase 1 resulting in interferon regulatory factor 3/7 activation (33); it controls NF-κB activation (34), and it ubiquitinates the NR2B subunit of NMDA receptors to down-regulate their activity (35). Our experiments using mutant ubiquitin, GABA_B_ receptor ubiquitination-deficient mutants, as well as *in situ* PLA indicate that MIB2 mediates Lys-63-linked ubiquitination of GABA_B1_ and thereby controls their lysosomal degradation.

To verify the importance of MIB2-mediated Lys-63-linked ubiquitination for lysosomal degradation, we tested its involvement in an experimental setting that mimics an important aspect of cerebral ischemia (sustained activation of glutamate receptors), which leads to a rapid down-regulation of GABA_B_ receptors via lysosomal degradation (16–19). Interestingly, prolonged activation of neurons with glutamate considerably increased the expression levels of MIB2 within 15–30 min. This rapid up-regulation of MIB2 might be enabled by the auto-ubiquitination activity of MIB2. The turnover of MIB2 has been suggested to be regulated by the interplay of its auto-ubiquitinating activity, leading to its proteasomal degradation, and the activity of interacting deubiquitinating enzymes (36). Thus, it is conceivable that sustained activation of glutamate receptors may increase the activity of an MIB2-associated deubiquitinase, which prevents auto-ubiquitination and proteasomal degradation of MIB2. The enhanced expression of MIB2 was accompanied by an increased interaction of MIB2 with GABA_B_ receptors and an elevated Lys-63-linked ubiquitination. Interfering with Lys-63-linked ubiquitination by overexpressing ubiquitin mutants or our GABA_B1a_(Lys → Arg) mutants prevented glutamate-induced down-regulation of the receptors. These results indicate that MIB2-mediated Lys-63-linked ubiquitination is indispensable for down-regulating the receptors via the lysosomal pathway and that the level of lysosomal degradation of the receptors is, at least in part, dependent on the expression level of MIB2.

In conclusion, our data suggest that MIB2-mediated Lys-63-linked ubiquitination of GABA_B1_ sorts GABA_B_ receptors to lysosomes for degradation under physiological as well as pathological conditions.

## Experimental Procedures

### 

#### 

##### Antibodies

The following antibodies were used: mouse anti-HA (1:1000 for immunofluorescence, 1:500 for *in situ* PLA, Sigma); rabbit GABA_B1b_ directed against the N terminus of GABA_B1b_ (affinity-purified, 1:200 for immunofluorescence, custom-made by GenScript) (37); rabbit GABA_B2_ directed against the N terminus of GABA_B2_ (affinity-purified, 1:500 for immunofluorescence; custom-made by GenScript) (38); guinea pig GABA_B2_ (1:500 for immunofluorescence; Millipore catalog no. AB2255, lot no. 2484228); mouse GABA_B1_ (1:100 for PLA; NeuroMab, clone N93A/49, catalog no. 7-183); rabbit ubiquitin Lys-48-specific (clone Apu2, 1:50 for *in situ* PLA; Millipore, catalog no. 05-1307, lot no. 2385989); rabbit ubiquitin Lys-63-specific (clone Apu3, 1:50 for *in situ* PLA; Millipore, catalog no. 05-1308, lot no. 2575910); and rabbit MIB2 (1:1000 for immunofluorescence, 1:250 for PLA; MyBiosource catalog no. MBS2014413, lot no. A20160407515). Secondary antibodies were purchased from Jackson ImmunoResearch labeled with either Alexa Fluor 488 (1:800), Cy-3 (1:500), or Cy-5 (1:300).

##### Drugs

The following chemicals were used for this study: glutamate (50 μm, Sigma) and leupeptin (100 μm, Sigma).

##### Plasmids

The following DNAs were used: HA-tagged GABA_B1a_ (39); GABA_B1a_(RSAR) (22); GABA_B2_ (40); HA-tagged ubiquitin (Addgene plasmid 17608); HA-tagged ubiquitin (KO) (Addgene plasmid 17603); HA-tagged ubiquitin Lys-63 (Addgene plasmid 17606); and HA-tagged ubiquitin K48R (Addgene plasmid 17604) (41). HA-tagged ubiquitin K63R was kindly provided by L.-Y. Liu-Chen, Temple University, Philadelphia; wild-type EGFP-tagged Rab7 was from Addgene (plasmid 12605); the functionally inactive mutant EGFP-tagged Rab7(DN) was from Addgene (plasmid 12660) (42); and HA-tagged MIB2 was from Addgene (plasmid 33312) (34).

##### Mutation of GABA_B1_

Lysines 697, 698, 892, and 960 in GABA_B1a_ were mutated to arginines using the QuikChange II XL site-directed mutagenesis kit from Stratagene according to the manufacturer's instructions.

##### Culture and Transfection of Cortical Neurons

Primary neuronal cultures of cerebral cortex were prepared from 18-day-old embryos of Wistar rats as described previously (10). Neurons were used after 11–15 days in culture. Plasmid DNA was transfected into neurons by magnetofection using Lipofectamine 2000 (Invitrogen) and CombiMag (OZ Biosciences) as detailed previously (43).

##### Immunocytochemistry and Confocal Laser Scanning Microscopy

Immunofluorescence staining was performed as described previously (10, 44). For selective detection of cell surface GABA_B_ receptors, living neurons were incubated with antibodies recognizing the extracellularly located N-terminal domain of GABA_B1_ or GABA_B2_ for 1 h at 4 °C. For analysis of total GABA_B_ receptors, neurons were fixed with 4% paraformaldehyde for 15–20 min at room temperature and permeabilized with 0.2% Triton X-100 before immunostaining.

Stained neurons were analyzed by laser scanning confocal microscopy (LSM 510 Meta or LSM 700, Zeiss). Images of eight optical sections spaced by 0.3 μm were recorded with a ×100 plan-Fluar oil differential interference contrast objective (1.45 NA, Zeiss) at a resolution of 1024 × 1024 pixels. Quantitative analysis of total and cell surface staining was performed as described previously (44).

##### In-cell Western Assay

Total GABA_B_ receptor expression of neurons cultured in 96-well plates was analyzed using the in-cell Western assay exactly as described previously (17). Fluorescence signals generated by GABA_B1_ and GABA_B2_ antibodies were normalized to actin signals determined simultaneously in the same cultures.

##### In Situ PLA

*In situ* PLA is an antibody-based technology for the detection of protein-protein interactions and post-translational modifications of proteins in cells *in situ* (45, 46). The *in situ* PLA was performed using Duolink PLA probes and detection reagents (Olink Bioscience, Sigma) according to the manufacturer's instructions as described previously (44). Here we applied *in situ* PLA primarily for the detection and quantification of GABA_B_ receptor ubiquitination using mouse GABA_B1_ or mouse HA antibodies together with rabbit antibodies specifically detecting Lys-48-linked or Lys-63-linked ubiquitin. Quantification was done by counting individual *in situ* PLA spots using the Mac Biophotonics ImageJ software. The number of spots was normalized to the area analyzed and to the expression level of GABA_B_ receptors.

##### Statistics

The statistical analyses were done with GraphPad Prism 5. The tests used and *p* values are given in the figure legends. Differences were considered statistically significant when *p* < 0.05.

## Author Contributions

K. Z. conceived and conducted most of the experiments, analyzed the data, and contributed to writing the manuscript. C. T. conducted and analyzed the experiments shown in Figs. 3*B*, 4, and 5. D. B. conceived the project, analyzed the data, and wrote the manuscript.

## Footnotes

2 The abbreviations used are:

ER: endoplasmic reticulum
ERAD: ER-associated degradation
Ub: ubiquitin
ANOVA: analysis of variance
PLA: proximity ligation assay
EGFP: enhanced GFP.
